# Efficacy of artemisinin-based combination therapy (ACT) in people living with HIV (PLHIV) diagnosed with uncomplicated *Plasmodium* falciparum malaria in Africa: a WWARN systematic review

**DOI:** 10.1186/s12936-025-05393-8

**Published:** 2025-05-16

**Authors:** Abena Takyi, Aboubakar Soma, Marianna Przybylska, Eli Harriss, Karen I. Barnes, Prabin Dahal, Philippe J. Guérin, Kasia Stepniewska, Verena I. Carrara

**Affiliations:** 1 Department of Child Health, Korle Bu Teaching Hospital, Accra, Ghana; 2 Centre for Tropical Medicine and Global Health, Nuffield Department of Medicine, University of Oxford, Oxford, UK; 3 Infectious Diseases Data Observatory (IDDO), Oxford, UK; 4 WorldWide Antimalarial Resistance Network (WWARN), Oxford, UK; 5 Centre MURAZ/Institut National de Santé Publique (INSP), Bobo-Dioulasso, Burkina Faso; 6 Département Médecine-Pharmacopée Traditionnelle/Pharmacie (MEPHATRA/Ph), Institut de Recherche en Sciences de la Santé (IRSS), Ouagadougou, Burkina Faso; 7 Royal Infirmary Edinburgh, NHS Lothian, Edinburgh, UK; 8 Bodleian Health Care Libraries, University of Oxford, Oxford, UK; 9 Division of Clinical Pharmacology, Department of Medicine, University of Cape Town, Cape Town, South Africa; 10 Institute of Global Health, Faculty of Medicine, University of Geneva, Geneva, Switzerland

**Keywords:** Human immunodeficiency virus, Anti-retroviral drugs, People living with HIV, Artemisinin-based combination therapy, Malaria, Drug-drug interactions

## Abstract

**Background:**

Africa bears the highest double burden of HIV and malaria worldwide. In 2023, an estimated 25.9 million people were living with HIV (PLHIV), and 246 million malaria cases were diagnosed in Africa. Malaria patients co-infected with HIV are considered at a higher risk of failing malaria treatment, according to the World Health Organization (WHO) guidelines. This systematic literature review aims to assess the treatment outcomes following artemisinin-based combination therapy (ACT) in PLHIV.

**Methods:**

The literature search was conducted up to April 2022 in the following databases: MEDLINE, EMBASE, Web of Science, Cochrane Central, WHO Global Index Medicus, Clinicaltrials.gov, and the WorldWide Antimalarial Resistance Network (WWARN) Clinical Trial Library. Studies describing any malaria treatment outcomes or anti-malarial drug exposure in PLHIV treated for uncomplicated *Plasmodium falciparum* malaria infection were eligible for inclusion.

**Results:**

A total of 26 articles describing 19 studies conducted between 2003 and 2017 in six countries were included in this review; it represented 2850 malaria episodes in PLHIV across various transmission settings. The most studied artemisinin-based combination was artemether-lumefantrine (in 16 studies). PLHIV were treated with various antiretroviral therapy (ART) regimens, namely efavirenz (EFV), nevirapine (NVP), atazanavir-ritonavir (ATVr), lopinavir-ritonavir (LPV/r), and/or on prophylaxis with trimethoprim-sulfamethoxazole (TS), or were untreated (in 3 studies). There was no evidence of an increased risk of recrudescence in PLHIV compared to those without HIV. When treated with artemether-lumefantrine, PLHIV receiving LPV/r had a lower risk of malaria recurrence compared to PLHIV on NVP-based or EFV-based ART, or those without HIV. LPV/r increased lumefantrine exposure and EFV-treated patients had a reduced exposure to both artemether and lumefantrine; NVP reduced artemether exposure only.

**Conclusions:**

Limited data on ACT outcomes or drug exposure in PLHIV in Africa remains a reality to date, and the effect of antivirals appears inconsistent in the literature. Considering the heterogeneity in study designs, these review’s findings support conducting an individual patient data meta-analysis to explore the impact of antiretroviral therapy on anti-malarial treatment.

*Trial registration*: The protocol for the original search was published on PROSPERO with registration number CRD42018089860.

**Supplementary Information:**

The online version contains supplementary material available at 10.1186/s12936-025-05393-8.

## Background

The African continent bears the highest double burden of HIV and malaria worldwide. In 2023, an estimated 25.9 million people lived with HIV (PLHIV) in Africa (65% of all PLHIV) [1], and 246 million malaria cases were diagnosed in the World Health Organization (WHO) African Region, or 93.5% of all cases worldwide [2]. A recent publication estimated that in 2020, 1.7 to 2.2 million PLHIV living in 41 African countries may suffer from uncomplicated *Plasmodium falciparum* malaria, contributing to 1.2% of all estimated uncomplicated *P. falciparum*, malaria cases in this region [3].

Naturally acquired immunity against malaria is only partial, and consequently, the immune system of PLHIV residing in malaria-endemic countries has to contend with both HIV and potentially multiple episodes of malaria. As the virus suppresses this acquired partial immunity [4], adult PLHIV may suffer from more frequent symptomatic malaria infections in areas of moderate to high malaria endemicities [5] and are more likely to experience severe disease in low-transmission areas [6]. HIV infection has also been shown to increase the risk of malaria infection and has been associated with higher parasite density in pregnancy [7], with a greater risk of malaria re-infection, and treatment failure in adults, even in high transmission areas [5].

Ensuring the efficacy of anti-malarial drugs in a high-risk population, such as PLHIV, is therefore paramount. However, trials specifically studying this co-infection are scarce. A systematic review published in 2011 identified 10 studies that investigated the impact of HIV on anti-malarial treatment response, of which there were only 3 studies that evaluated artemisinin-based combination therapy (ACT) [8]. The review found that HIV infection is associated with increased prevalence and severity of clinical malaria and was also associated with impaired response to anti-malarial treatment that was dependent on age, immunosuppression, and previous immunity to malaria. No recent systematic review on efficacy of ACT, the current mainstay of anti-malarial treatment, has been conducted in this population.

Since HIV requires life-long treatment with Highly Active Antiretroviral Therapy (HAART), drug-drug interaction(s) with anti-malarials present possible complications in management of malaria-HIV co-infection. HAART aims to boost the immunity of PLHIV [9]. Hence, it is expected that immunity against malaria should also improve. However, the pharmacokinetic properties of anti-malarial drugs may be affected by the presence of HAART, which can alter drug exposure [10, 11]. Adverse effects of interactions between ACT and some HAART on liver function and bone marrow suppression have been reported previously [12]. Similarly, some drug-drug interactions were previously reported for TS prophylactic treatment, which increases protection against malaria and other opportunistic infections in PLHIV. However, systematic evidence for the effect of drug-drug interactions between ACT and HAART on anti-malarial treatment efficacy is lacking.

The objective of this systematic review was to estimate the efficacy of anti-malarial treatment for uncomplicated *P. falciparum* infection in PLHIV in Africa and compare it with efficacy in HIV-uninfected patients.

## Methods

### Search strategy

The initial search was conducted on 02/09/2019 by a librarian (EH) at the Bodleian Health Care Libraries, University of Oxford, which included all studies published until the search date. The following databases were searched: Ovid MEDLINE, Ovid EMBASE, Web of Science (all Databases), Cochrane Central Register of Controlled Trials, WHO Global Index Medicus and Clinicaltrials.gov. Updates of the search were conducted on 30/10/2020, 01/07/2021, and 28/04/2022 as part of the WWARN Clinical Trial Library [13] and all additional studies published from 01/01/2019 were screened for inclusion. No restrictions were placed on language or publication date. The full list of search terms is available in Additional File 1 (initial search) and Additional File 2 (searches in WWARN Clinical Trial Library). Briefly, search terms used in the search strategy included: “Malaria”, “malaria.ti,ab.”, “Plasmodium,” “plasmodium.ti,ab.”, “falciparum,” “Africa,” the name of each African country, or"Central* Africa*"or"West* Africa*"or ‘‘East* Africa*’’ or ‘‘North* Africa*’’ or ‘‘South* Africa*’’ or ‘‘sub Saharan Africa*’’ or ‘‘sub-Saharan Africa,*” “artemisinin,” “artemisinin derivative,” the names of each individual component of the ACT, “human immunodeficiency virus infection” or “acquired immune deficiency syndrome” and other related terms.

### Inclusion and exclusion criteria

PLHIV of all ages diagnosed with confirmed uncomplicated *P. falciparum* malaria in Africa were included. Since not every PLHIV has access to life-saving HAART despite its availability for over two decades in sub-Saharan Africa [1], patients on any antiretroviral therapy (ART), TS prophylactic treatment and those not yet on treatment were included. Patients with asymptomatic parasitaemia, severe malaria or unconfirmed malaria were excluded.

The following artemisinin-based combinations were included in this review: artemether-lumefantrine (AL); artesunate-amodiaquine (ASAQ); artesunate-mefloquine (ASMQ); artesunate-sulfadoxine-pyrimethamine (ASSP); dihydroartemisinin-piperaquine (DP); artesunate-pyronaridine (AP).

Studies included were randomized control trials (RCTs), quasi-randomized controlled trials, case–control studies, and longitudinal cohort studies. Pharmacokinetic studies were also included. Animal studies, prevention studies, case reports/case series, retrospective studies, systematic reviews, and literature reviews were excluded.

### Study outcomes and data extraction

The primary outcome was polymerase chain reaction (PCR) adjusted treatment failure (recrudescence) of ACT, as defined by the WHO, on day 28 of treatment for PLHIV [14]. Secondary outcomes included other measures of treatment failure such as: PCR confirmed reinfection, recurrence, early treatment failure as well as outcomes recorded on days 42 or 63 of follow-up [14, 15]. Pharmacokinetic parameters, if available specifically for PLHIV, were also extracted.

Two reviewers (AT and MP, or AS and MP) independently assessed the eligibility of studies by screening the title and abstract and conducting full text screening of selected studies. Studies were excluded at the title and abstract screening stage if there were no HIV cases, no confirmed uncomplicated *P. falciparum* malaria cases, study sites were not in Africa, prevalence studies, prevention studies, treatment did not include ACT or studies with inappropriate study design. Disagreements between reviewers were resolved by the third reviewer (KS).

Data extracted included study year, site, design, inclusion and exclusion criteria for patients, number of enrolled patients, treatment regimens, number of patients treated with each regimen, and reported outcome. For each reported outcome and each arm/patient subgroup, number of patients evaluated, day of assessment and treatment efficacy results were extracted. When provided, measurements of treatment differences in anti-malarial treatment efficacy, such as Hazard Ratios (HR), Risk Ratio (RR) or Odds Ratio (OR) with 95% confidence intervals (CI), and p-value were extracted. Any estimates of pharmacokinetics (PK) parameters for anti-malarial drugs from pharmacokinetic studies were included.

### Risk of bias assessment

Risk of bias in individual studies was assessed using the Cochrane tools RoB 2 for randomized studies [16] and ROBINS-I for non-randomized studies [17]. A set of signalling questions was used to make a judgement on the likely extent of bias for each of the studies across different domains under consideration (see Additional Files 3 and 4 for a full set of signalling questions and judgments for each of the studies). Certainty of evidence for each outcome was assessed according to the GRADE guidelines [18].

### Statistical analysis

Due to the small number of studies and limited information provided in publications, only descriptive analysis was carried out for the majority of outcomes. Meta-analysis could only be conducted to compare lumefantrine concentrations on day 7 between different ART regimens and to compare artemether, or its metabolite dihydroartemisinin, exposure between PLHIV treated with NVP and HIV-uninfected patients. Fixed effect models using the method of Mantel and Haenszel were fitted and I-squared was used as a relative measure of heterogeneity between studies.

Since studies included in this review were conducted in high or moderate transmission intensity areas, efficacy estimates were presented for recrudescence only, while relative estimates (OR, HR) were presented for any outcomes for comparison between PLHIV and HIV-uninfected patients, or different ART regimens in PLHIV. Where raw data were available, the calculations were conducted to estimate proportion (95% CI) of patients with the outcome of interest; OR (95% CI) were calculated from proportions, and HR were calculated from the Kaplan–Meier (KM) estimates as outlined in Klein et al*.* [19]. Interquartile ranges (IQR) of the drug levels, if not provided, were estimated from other reported parameters such as range (after logarithmic transformation, using a method outlined in Hozo et al. [20] and implemented in an online calculator [21]), or from reported mean and its 95% CI (assuming normal distribution). Similarly, mean and 95% CI were estimated from median and IQR as proposed by Wan et al*.* [22].

## Results

**Fig. 1:**
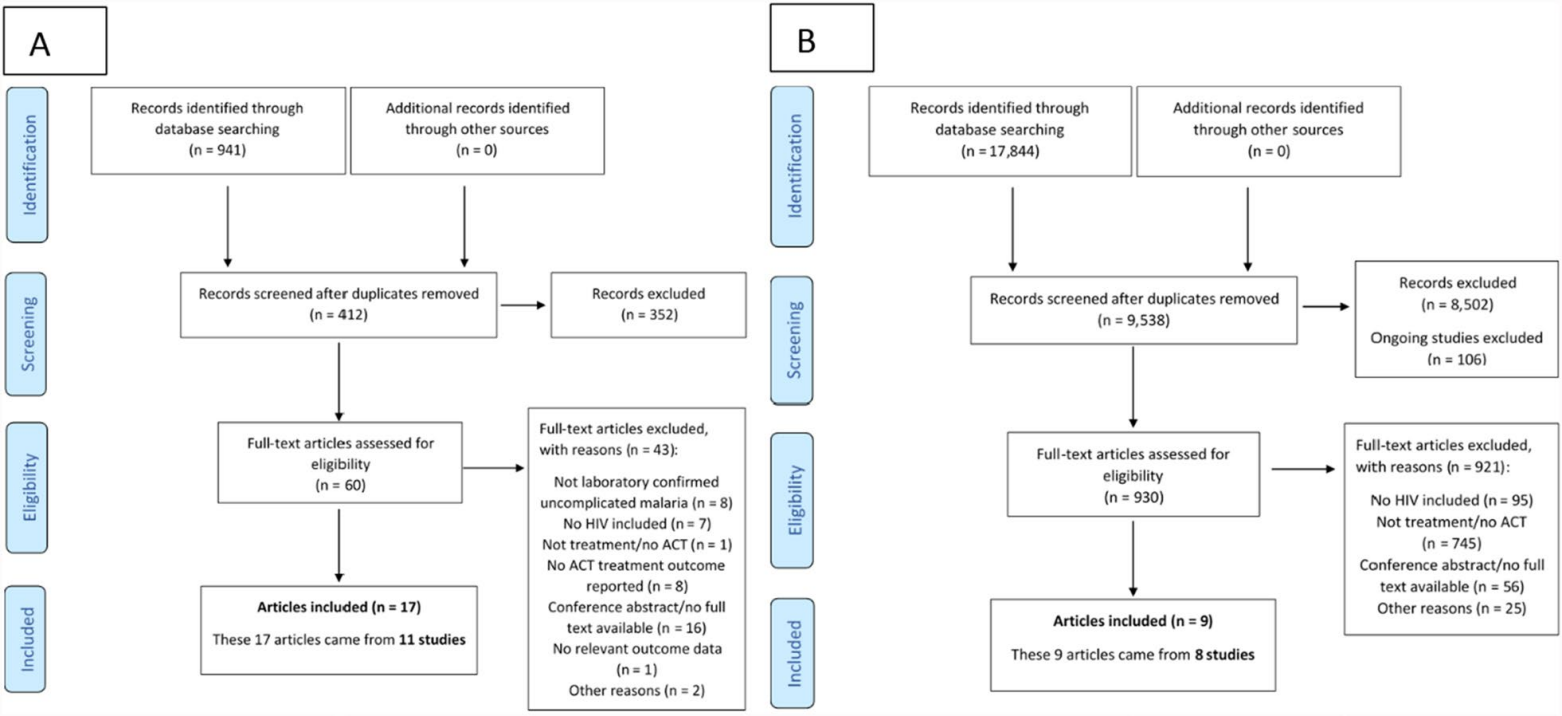
PRISMA profile for systematic review. On the left (panel **A**), initial search on MEDLINE, EMBASE, Web of Science (all Databases), Cochrane Central, WHO Global Index Medicus and Clinicaltrials.gov. On the right (panel **B**), subsequent searches performed in the WWARN Clinical Trial Library

A total of 9950 articles were identified for screening, 990 were included in the full text screening (Fig. 1). Twenty-six articles (originating from 19 studies) were identified for inclusion in the review. These studies were conducted between 2003 and 2017 in various endemicity areas in Uganda (n = 10), Nigeria (n = 4), Zambia (n = 2) and Tanzania (n = 1). There were two multi-country studies (one in Malawi and Uganda, and the other in Malawi and Mozambique) (Table 1).

**Table 1:** Summary of the 26 articles included in the review and their associated study (Study ID)

Study ID	First author, date and PMID of all included articles	Study years	Country	Transmission intensity	Study design	Patients enrolled	Age (years)	Pregnancy included	HIV-uninfected Included	Malaria drug	HIV drug	Total malaria episodes in PLHIV	Total malaria episodes overall	Clinical study	PCR	PK parameters
1	Huang-2017; 29,065,172 [40]	2012–2014	Malawi Uganda	Not provided	Observational, non-randomized study	39	3.6–12	N/A	Yes (historical controls)	AL	NVP	15	35	No	N/A	Artemether DHA LUM
2	1. Maganda-2014; 24,885,714 [25] 2. Maganda-2015, 25,906,774 [38] 3. Maganda-2016; 25,963,334 [58]	2010–2012	Tanzania	Moderate	Open-label, parallel three-arm study	269	19–67	No	No	AL	EFV NVP None	269	269	Yes	No	LUM
3	Gasarira-2008; 18,444,813 [45]	2005–2006	Uganda	Moderate	Longitudinal, 2 prospective cohorts	293	1–10	N/A	Yes	ASAQ	TS + ART	35	293	Yes	Yes	No
4	Sambol-2015; 25,732,044 [39]	2008	Uganda	High	Part of longitudinal, open-label RCT	107	0.5–2	N/A	Yes	DP	TS + ART	12^c^	218	No	N/A	PPQ
5	1. Arinaitwe-2009; 19,877,969 [59] 2. Verret-2011; 21,383,095 [29]	2007–2009	Uganda	High	Longitudinal, open-label RCT	292	0.33–3	N/A	Yes	AL DP	TS TS + ART	172	2013	Yes	Yes^a^	No
6	1. Kakuru-2013; 23,382,157 [36] 2. Wanzira-2014; 24,825,870 [30]	2007–2011	Uganda	High	Longitudinal, open-label RCT	312	0.33–5	N/A	Yes	AL DP	TS + ART	44^d^	5564	Yes	No	No
7	Muhindo-2014; 24,468,007 [35]	2011–2012	Uganda	High	Longitudinal, open-label RCT	202	4–5	N/A	Yes	AL DP	TS + ART	38	770	Yes	No	No
8	Kakuru-2014; 24,759,826 [31]	2009–2013 2007–2012	Uganda	High	Longitudinal, 2 open-label RCTs	166	0.12–6	N/A	No	AL DP	EFV NVP LPV/r None	938	938	Yes	No	No
9	Achan-2012; 23,190,222 [33]	2009–2011	Uganda	High	Longitudinal, open-label RCT	170	0.5–7	N/A	No	AL	EFV NVP LPV/r	281	281	Yes	No	LUM
10	Musoke-2012; ISSN: 1816–3319 [37]	2010–2011	Uganda	High	Open-label, parallel three-arm study	36	Adults	No	No	AL	TS TS + EFV	36	36	No	N/A	LUM
11	1. Kajubi-2016; 28,018,925 [34] 2. Parikh-2016; 27,143,666 [26]	2011–2014	Uganda	High	Longitudinal, prospective PK/PD study	252	1.1–8.6	N/A	Yes	AL	EFV NVP LPV/r	118	134	Yes	Yes	Artemether DHA LUM
12	1. Van Geertruyden-2006; 16,960,779 [24] 2. Van Geertruyden-2009; 19,922,664 [60]	2003–2005	Zambia	Moderate	RCT	970	Adults	No	Yes	AL SP	None	320	970	Yes	Yes	No
13	Usman-2021; 33,755,736 [61]	Not provided	Nigeria	Not provided	PK parallel study	20	Adults ≥ 18	No	Yes	AL	ATV/r	10	20	No	N/A	LUM
14	1. Adegbola-2018; 30,082,286 [62] 2. Adegbola-2020; 32,209,837 [63]	2016–2017	Nigeria	Unstable	PK parallel study	69	18–45	Yes	No	AL	EFV	52	52	No	N/A	LUM Desbutyl-Lum
15	Hugues-2020; 31,929,402 [28]	2012–2014	Uganda	High	PK parallel study	39	≥ 16	Yes, all	Yes	AL	EFV	39	39	Yes	No	Artemether DHA LUM
16	Usman-2020; 32,921,396 [54]	Not provided	Nigeria	Not provided	PK parallel study	40	≥ 18	No	Yes	AL	EFV NVP LPV/r	30	40	No	N/A	LUM
17	Banda-2019; 31,126,288 [64]	2014–2015	Zambia	Moderate-High	Single-arm interventional study	152	15–65	No	No	AL	EFV	152	152	Yes	Yes	No
18	Sevene-2019; 31,429,785 [32]	2013–2015	Malawi Mozambique	Moderate-High	Single-arm interventional study	221	15–65	No	No	DP	EFV NVP	221	221	Yes	Yes	No
19	Chijioke-Nwauche-2013; 23,774,430 [27]^b^	2010–2011	Nigeria	Holoendemic	PK parallel study	167	16–65	Not provided	Yes	AL	EFV NVP	68	167	Yes	No	LUM

Study 8 combines a set of patients from Study 4 and an extension of Study 8 is reported in Study 9. Studies will be referred to in the text (i.e. ID 1) and the relevant article afferent to the study will be referenced

*ACT* artemisinin combination therapy, *AL* artemether-lumefantrine, *ART* antiretroviral therapy, *ASAQ* artesunate-amodiaquine, *ATV/r* atazanavir-ritonavir, *Desbutyl-Lum* desbutyl-lumefantrine, *DHA* dihydroartemisinin, *DP* dihydroartemisinin-piperaquine, *EFV* efavirenz, *LUM* lumefantrine, *LPV/r* lopinavir/ritonavir, *N/A* information not available, *NVP* nevirapine, *PLHIV* people living with HIV, *PK* pharmacokinetics parameters; PCR: polymerase chain reaction (adjusted treatment failure), *PPQ* piperaquine, *SP* sulfadoxine-pyrimethamine, *TS* trimethoprim-sulfamethoxazole prophylactic treatment

^a^PCR done, but no PCR corrected results presented

^b^All malaria slides positive by microscopy, but 78.1% were aparasitaemic by nested-PCR

^c^12 children were HIV-infected and had at least one malaria episode; total number of episodes not reported

^d^44 children were HIV-infected and had at least one malaria episode; total number of episodes not reported

The artemisinin-based combinations studied included AL in 16 studies (including 10 pharmacokinetic studies of lumefantrine, and of artemether and its metabolite dihydroartemisinin in 3 of them), DP in six studies (5 clinical and one study reporting piperaquine pharmacokinetics) and ASAQ in one clinical study. In total, 12,450 malaria episodes were reported in the included studies, with 79% of the episodes (n = 9784/12,450) reported in longitudinal cohorts of children studied in Tororo, Uganda (study ID 4–9, Table 1). There were 2850 malaria episodes among PLHIV.

Seven studies (5 in adults and 2 in children) included only PLHIV; two studies included pregnant women. Eight studies compared malaria outcomes under different ART regimens, namely efavirenz (EFV), nevirapine (NVP), lopinavir-ritonavir (LPV/r) or atazanavir-ritonavir (ATV/r), and/or prophylaxis with TS. Detailed description of HIV-related inclusion criteria is available in Additional File 5. Most studies did not have restrictions on the CD4 count in PLHIV at enrolment, and only seven studies provided a baseline CD4 count: in those, PLHIV were on ART for at least two weeks prior to enrolment with a reported median CD4 count > 200 cells/mm^3^ or a CD4 percentage > 20% [23].

### Late treatment failure

Eleven studies presented findings regarding late treatment failure. Risk of recurrence was compared between malaria patients with and without HIV infection in seven studies (Table 2).

**Table 2:** Comparison of anti-malarial treatment efficacy between PLHIV and HIV-uninfected malaria patients

Study ID	Malaria drug	HIV drug	N: HIV-	Event HIV-	N: HIV +	Event HIV +	Day	Statistics	Type of analysis	Comparison HIV (−) to HIV (+) Estimate	95%CI	P-value
Recrudescence												
3	ASAQ	TS, ART^a^	250	9^b^	34	0	28	HR^c^	Univariable	N/A		0.25
11	AL	EVF + LPV/r + NVP	181	5^d^	181	6^d^	28	OR^e^	Univariable	0.83	0.20–3.33	1
12	AL or SP	None	530	61	266	37	45	RR	Multivariable controlled for treatment	0.85	0.58–1.23	0.38
Recurrence												
3	ASAQ	TS, ART	250	33	34	1	28	HR^2^	Univariable	1.75		0.08
5	DP	TS, NVP^f^	705	N/A	276	N/A	42	HR	Univariable	1.75		0.001
	AL	TS, NVP^f^	699	N/A	333	N/A	42	HR	Univariable	1.51		0.002
11	AL	EFV	181	88	50	19	28	OR	Multivariable controlled for day 7 lumefantrine concentration	2.84	1.04–7.78	0.04
	AL	EFV	181	88	50	19	28	OR	Multivariable	1.73	0.73–4.11	0.21
	AL	EFV	181	88	50	19	28	OR	Univariable	1.87	0.86–4.06	0.11
15	AL	EFV	30	4	9	3	42	OR	Univariable	0.31	0.05–1.75	0.319
11	AL	LPV/r	181	88	70	10	28	OR	Multivariable controlled for day 7 lumefantrine concentration	5.03	1.58–15.98	0.006
	AL	LPV/r	181	88	70	10	28	OR	Multivariable	6.48	2.18–19.21	0.0008
	AL	LPV/r	181	88	70	10	28	OR	Univariable	6.32	2.23–17.95	0.0005
	AL	NVP	181	88	61	19	28	OR	Multivariable controlled for day 7 lumefantrine concentration	2.22	1.10–4.48	0.03
	AL	NVP	181	88	61	19	28	OR	Multivariable	2.27	1.12–4.60	0.02
	AL	NVP	181	88	61	19	28	OR	Univariable	2.36	1.21–4.58	0.01
19	AL	NVP (1 EFV)^g^	99	12	68	12	28	OR	Univariable	0.64	0.27–1.53	0.317
12	AL or SP	None	530	86	266	49	45	RR	Multivariable controlled for treatment	0.89	0.65–1.22	0.45
6	AL	TS, ART^h^	2371	774	533	128	28	HR^b^	Univariable	1.55		NA
	DP	TS, ART^h^	2176	153	484	29	28	HR^b^	Univariable	1		NA
	AL	TS, ART^h^	2371	N/A	533	N/A	42	HR^b^	Univariable	1.9		NA
	DP	TS, ART^h^	2176	N/A	484	N/A	42	HR^b^	Univariable	1.18		NA
	AL	TS, ART^h^	2371	N/A	533	N/A	84	HR^b^	Univariable	2.02		NA
	DP	TS, ART^h^	2176	N/A	484	N/A	84	HR^b^	Univariable	2.07		NA
New infection												
12	AL or SP	None	530	25	266	12	45	RR	Multivariable controlled for treatment	1.04	0.53–2.04	0.89

*AL* artemether-lumefantrine, *ART* antiretroviral therapy, *ASAQ* artesunate-amodiaquine, *DP* dihydroartemisinin-piperaquine, *EFV* efavirenz, *HR* hazard ratio, *LPV/r* lopinavir/ritonavir, *N/A* information not available, *NVP* nevirapine, *SP* sulfadoxine-pyrimethamine, *TS* trimethoprim-sulfamethoxazole prophylactic treatment, *OR* odds ratio, *RR* relative risk

^a^Children meeting WHO eligibility criteria received ART (Zidovudine)

^b^Calculated from KM estimates, HR estimated using the formula by Klein et al*.* [19]. KM estimates either provided in text or extracted from the figure

^c^Could not be calculated as no recurrences were observed in the PLHIV group

^d^Based on genotyping results for 96% of recurrences, assuming recurrences with missing PCR were reinfections

^e^Calculated from raw numbers provided in the article, assuming 4% of recurrences with missing PCR were reinfections

^f^Includes PLHIV and patients exposed to HIV (DP: 71 infected, 64 on NVP; AL: 101 infected, 94 on NVP)

^g^All PLHIV were identified through attendance at a weekly clinic in which all received NVP (except for a single patient on efavirenz; when this patient was excluded from the analysis, the relationship between NVP use and lumefantrine concentration on day 7 remained significant)

^h^TS includes also patients exposed to HIV who were randomly assigned to TS

One study (ID 12) conducted in Zambian adults investigated malaria efficacy in PLHIV not yet on ART. This study treated malaria with AL or SP, and presented results for the combined treatment arms on day 45 [24]; after adjusting for treatment, there were no significant differences between PLHIV and HIV-uninfected patients with respect to risk of recurrence, recrudescence or reinfection. However, among PLHIV, the risk of malaria treatment failure (unadjusted for recrudescence or reinfection) on day 45 was found to be 2.24-fold higher among those with CD4 cell count < 300 cells/µL compared to those with CD4 cell count ≥ 300 cells/µL (RR 2.24, 95%CI 1.20–4.17, p-value = 0.01). This finding was not confirmed in the other study (ID 2) as no significant difference in risk of recurrence at day 28 was observed between patients with CD4 cell count above or below 350 cells/µL [25]. Our search identified only one study from Uganda conducted in children (ID 11), which explored the effect of HIV and different ART treatments on malaria treatment outcomes. This study found the risk of recurrence in children living with HIV on ART was significantly lower than in children who were HIV-uninfected [26]. The overall odds ratio (OR) of recurrence (adjusted for day 7 lumefantrine concentration) among the HIV-uninfected group was higher compared to PLHIV on LPV/r, NVP or EFV-based regimens, and these were 5.03 (1.58–15.98, p = 0.006), 2.22 (1.10–4.48, p = 0.003) and 2.84 (1.04−7.78, p = 0.04) respectively [26]. When recurrence was compared among the 3 ART regimens, children on EFV had an adjusted OR (AOR) 3.74 times that of LPV/r. A similar recurrence was observed between LPV/r and NVP. In children treated with EFV, the observed frequency of recurrence was similar to the HIV-uninfected group and this was due to lower lumefantrine drug exposure. The comparisons between all treatment groups were not significantly different for recrudescence. Two further studies in adults treated with AL found a higher proportion of recurrence in PLHIV receiving EFV (ID 15 in Uganda) or NVP (ID 19 in Nigeria), compared to HIV-uninfected patients; however, the differences were not significant [27, 28].

Three other studies (ID 3, ID 5, ID 6) compared outcomes in PLHIV on TS prophylaxis (some of whom were also on ART as per country protocols) to HIV-uninfected patients. These three studies showed consistent results of higher risk of recurrence in HIV-uninfected patients with corresponding HR on day 28 of 1.5 for AL and 1.75 for ASAQ, with a pooled OR of 1.35 (95% CI 1.16–1.56, p < 0.001, I^2^ = 0.0%, chi-square test for heterogeneity = 0.376), based on available observed proportions (ID 3, ID 6, AL or ASAQ) and ignoring losses to follow-up (as no information was available). For patients treated with DP, no difference (HR = 1.75) was observed until day 42 in the study by Verret et al*.* [29], and an estimated HR of 1.2 (derived from KM curves) was reported in the study by Wanzira et al*.* [30]. The HR reached 2 by day 84 in the study by Wanzira et al*.* [30]. Confidence intervals for HR were not provided or could not be calculated, and KM estimates could not be pooled as the standard errors were not available.

Table 3 presents the reported estimates of PCR-corrected risk of recrudescence in PLHIV in five studies. Estimates for recurrence are not provided as they are driven by the malaria transmission intensity which is moderate to high in all study sites (Additional File 6).

**Table 3:** Estimated PCR-corrected risk of recrudescence in PLHIV co-infected with malaria

Study ID	Day	Malaria drug	HIV drug	PLHIV group		%	95%CI	HIV-uninfected comparator		%	95%CI
				N	n			N	n		
3	28	ASAQ	TS + ART	35	0	0.0	0–10.0	258		3.6^a^	1.9–6.9^a^
11	28	AL	NVP	61	3	4.9	1.0–13.7	181	5	2.8	1.2–6.3
	28	AL	EFV	50	1	2.0	0.1–10.6	181	5	2.8	1.2–6.3
	28	AL	LPV/r	70	2	2.9	0.3–9.9	181	5	2.8	1.2–6.3
12	45	AL or SP	None	266	37	13.9	10.0–18.7	530	61	11.5	8.9–14.5
17	42	AL	EFV	134^b^	0	0.0	0.0–2.8				
18	42	DP	EFV	158^c^	0	0.0	0.0–2.4				
	42	DP	NVP	61	0	0.0	0.0–6.9				

N = number of patients enrolled, and n = number of failures. If only n/N were provided in the publication, proportion and 95% CI for proportion were calculated (Wilson method) assuming no losses to follow-up

*AL* artemether-lumefantrine, *ART* antiretroviral therapy, *ASAQ* artesunate-amodiaquine, *DP* dihydroartemisinin-piperaquine, *EFV* efavirenz, *LPV/r* lopinavir/ritonavir, *NVP* nevirapine, *SP* sulfadoxine-pyrimethamine, *TS* trimethoprim-sulfamethoxazole prophylactic treatment

^a^KM estimates are provided as reported in the publication

^b^Excludes 10 lost to follow-up and 8 with PCR results indeterminate or unavailable

^c^Excludes one early treatment failure and one with PCR result not available

Five studies (ID 2, ID 8, ID 9, ID 11, ID 18) compared the risk of recurrence after ACT in PLHIV on different ART regimens (Table 4). A slightly higher proportion of recurrent malaria was reported in patients on EFV compared to patients on NVP by day 28 [26, 31] or day 42 [32]; however, none of the comparisons were statistically significant. Compared to LPV/r, NVP and EFV had approximately threefold higher risk of recurrence by day 28 [26, 31, 33]; this finding was no longer apparent once the comparison was adjusted for lumefantrine concentration on day 7 in study ID 11 [26]. Study ID 9 [33] showed that lower concentrations of lumefantrine on day 7 were observed in EFV or NVP treatment groups compared to LPV/r (further discussed in the pharmacokinetic section). Meta-analysis was not attempted as raw data was either not available or different effect measures (OR, HR) were reported.

**Table 4:** Comparison of risk of recurrence of parasitaemia in PLHIV on different antiretroviral therapies or not yet on treatment

Study ID	ACT	ART Group 1	ART Group 2	N1	Events1	N2	Events2	Day	Statistics	Type of analysis	Estimate	95%CI	P-value
9	AL	NVP or EFV	LPV/r	86	N/A	84	N/A	63	HR	Univariable	2.44	1.32–4.54	0.004
	AL	NVP or EFV	LPV/r	86	N/A	84	N/A	28	HR	Univariable	3.23	1.47–7.14	0.004
2	AL	NVP	None	125	3	73	4	28	RR	Univariable	0.4	0.29–0.9	0.53
	AL	EFV	None	63	11	73	4	28	RR	Univariable	3.2	2.4–7.8	< 0.001
	AL	EFV	None	63	11	73	4	28	HR	Univariable	19.11	10.5–34.5	< 0.01
	AL	NVP	None	125	3	73	4	28	HR	Univariable	2.44	0.79–7.6	0.53
8	AL	EFV	NVP	102	N/A	410	N/A	28	HR	Multivariable controlled for age	1.76	0.99–3.13	0.06
	AL	NVP	LPV/r	410	N/A	249	N/A	28	HR	Multivariable controlled for age	2.65	1.27–5.26	0.009
	AL or DP	NVP	None	567	N/A	20	N/A	28	HR	Multivariable controlled for age	6.25	0.83–50.0	0.08
11	AL	EFV	LPV/r	50	19	70	10	28	OR	Univariable	3.38	1.01–11.35	0.49
	AL	EFV	LPV/r	50	19	70	10	28	OR	Multivariable	3.74	1.02–13.74	0.04
	AL	EFV	LPV/r	50	19	70	10	28	OR	Multivariable controlled for day 7 lumefantrine concentration	1,77	0.36–8.81	0.48
	AL	NVP	LPV/r	61	19	70	10	28	OR	Univariable	2.68	0.84–8.56	0.1
	AL	NVP	LPV/r	61	19	70	10	28	OR	Multivariable	2.86	0.85–9.60	0.09
	AL	NVP	LPV/r	61	19	70	10	28	OR	Multivariable controlled for day 7 lumefantrine concentration	2.26	0.64–8.05	0.21
	AL	EFV	NVP	50	19	61	19	28	OR	Univariable	1.26	0.5–3.17	0.62
	AL	EFV	NVP	50	19	61	19	28	OR	Multivariable	1.31	0.49–3.51	0.59
	AL	EFV	NVP	50	19	61	19	28	OR	Multivariable controlled for day 7 lumefantrine concentration	0.78	0.32–2.37	0.66
18	DP	EFV	NVP	159^a^	8	61	0	42	RD^b^	Univariable	5.0%	1.6–8.4	0.074

*ACT* artemisinin combination therapy, *AL* artemether-lumefantrine, *ART* antiretroviral therapy, *DP* dihydroartemisinin-piperaquine, *EFV* efavirenz, *HR* hazard ratio, *LPV/r* lopinavir/ritonavir, *N/A* information not available, *NVP*: nevirapine, *OR* odds ratio, *RR* relative risk

^a^Excludes 1 early treatment failure

^b^Risk difference (RD) calculated from proportions, not provided in the publication. Odds ratio (OR) cannot be calculated due to 0 events in one group

### Early parasitological response

Four studies (ID 7, ID 11, ID 12, ID 19) compared parasite clearance between PLHIV and HIV-uninfected patients (Table 5). In a study where patients were treated with AL or SP (ID 12), no significant difference in prevalence of parasite positive readings were found on day 3 [24], while in two studies (ID 7, ID 11) with patients treated with AL or DP, a significantly faster clearance was observed in HIV-uninfected patients [34, 35]. In study ID 11, median parasite clearance half-life was larger by 25% in PLHIV compared to HIV-uninfected patients (3.5 h versus 2.8 h) and this was consistent across the three HIV treatment groups (EFV, NVP and LPV/r) [34]. In study ID 19, Chijioke-Nwauche et al. showed similar proportion of patients with parasite positivity on day 3 between groups, but baseline parasitaemia, even though detected by microscopy, was undetectable by nested-PCR in 78.1% of patients [27].

**Table 5:** Summary of reported results for early parasitological response

Study ID	Malaria drug	HIV drug	Day	Statistics	PLHIV		HIV-uninfected group		Comparison		P-value
					Estimate	N	Estimate	N	Statistics	Estimate (95%CI)	
7	AL or DP	TS	1	Percentage positive	58.3		60.5		RR	0.97 (0.72–1.31)	0.84
	AL or DP	TS	2	Percentage positive	19.4		5.0		RR	3.87 (1.85–8.12)	< 0.001
9	AL	EFV or NVP	2	N (%) positive	9 (5.3)	171					
	AL	LPV/r	2	N (%) positive	9 (8.4)	107					
	AL	EFV or NVP	3	N (%) positive	2 (1.2)	171					
	AL	LPV/r	3	N (%) positive	2 (1.9)	105					
11	AL	EFV or NVP or LPV/r	3	Median (IQR) HL(h)	3.51 (2.98–4.03)	22	2.8 (2.38–3.36)	77			0.003
12	AL or SP	None	3	N/A		N/A		N/A	OR	1.15	0.6
17	AL	EFV	N/A	Median (range) PC50 (h)	5.7 (0.3–25.8)^a^	57					
	AL	EFV	N/A	Median (range) PC90 (h)	13.8 (2.9–32.9)^a^	57					
18	DP	EFV	N/A	Median (range) PC50 (h)	4.2 (0.6–40.3)	84					
	DP	EFV	N/A	Median (range) PC90 (h)	10.1 (3.2–63.1)	84					
	DP	EFV	N/A	Median (range) HL (h)	2.2 (1.2–9.8)	84					
	DP	EFV	N/A	N (%) HL > 5.5 h	5 (6.0%)	84					
	DP	NVP	N/A	Median (range) PC50 (h)	3.1 (0.2–10.3)	46					
	DP	NVP	N/A	Median (range) PC90 (h)	8.2 (2.4–19.7)	46					
	DP	NVP	N/A	Median (range) HL (h)	2.1 (1.1–6.8)	46					
	DP	NVP	N/A	N (%) HL > 5.5 h	1 (2.2%)	46					
19^b^	AL	NVP, EFV	3	N (%) positive	8 (11.8)	68	12 (12.1)	99			0.944

*AL* artemether-lumefantrine, *ART *antiretroviral therapy, *DP* dihydroartemisinin-piperaquine; *EFV* efavirenz, *h* hour, *HL* half-life, *HR* hazard ratio, *IQR* interquartile range, *LPV/r* lopinavir/ritonavir, *N/A* information not available, *NVP* nevirapine, *OR* odds ratio, *PC* parasite clearance, *RR* relative risk, *SP* sulfadoxine-pyrimethamine, *TS* trimethoprim-sulfamethoxazole preventive treatment

^a^PC50 = 6.0 (0.3–24.4) h and PC90 = 13.1 (2.9–30.9) h in the per protocol population, n = 54

^b^At baseline all patients were malaria positive by microscopy, but 78.1% were aparasitaemic by nested-PCR

Three further studies (ID 9, ID 17, ID 18) reported the results for early parasitological response in PLHIV only, either on EFV or NVP treatment. Parasite clearance was faster in PLHIV receiving EFV and treated with AL, compared to those treated with DP; and within the DP treatment group (ID 18), PLHIV receiving NVP parasite clearance was faster compared to PLHIV receiving EFV, although formal comparison was not presented [32]. Parasite positivity on day 2 or 3 was similar between patients on NVP or EFV, compared to LPV/r in study ID 9 [33].

### Gametocytaemia

No quantitative analysis was possible. Three studies (ID 6, ID 11, ID 12) compared gametocyte carriage between PLHIV co-infected with malaria and HIV-uninfected patients. In study ID 6, a higher risk of developing gametocytes within 28 days follow-up was found in patients with TS prophylaxis versus no TS, RR = 1.76 (95%CI 1.29–2.40, p < 0.001) [36]. In the same study, after adjusting for TS, patients’ age, treatment arm and recurrent parasitaemia status, the risk was not different between PLHIV and HIV-uninfected patients, RR = 1.29 (95% CI 0.74–2.24, p = 0.373) [36]. TS prophylaxis was also associated with delayed gametocyte clearance, with HR = 1.32 (95%CI 1.05–1.64, p = 0.02) compared to patients without TS prophylaxis.

In study ID 11, the proportion of episodes in which patients developed gametocytes after treatment (days 1–28) was 17% (of 188 episodes) in HIV-uninfected group compared to 20% (of 70 malaria episodes), 29.6% (62 malaria episodes) and 36% (in 50 episodes) in LPV/r, NVP, EFV treated groups of PLHIV (p = 0.008 for comparison between HIV-uninfected and a combined group treated with NVP or EFV) [26] respectively. In the study ID 12, presence of gametocytes on days 3, 7, 14, 28, 45 was not significantly different between HIV-uninfected patients and PLHIV not yet on treatment [24].

The two ART regimens (NVP or EFV, LPV/r) in the study ID 9 were not different in respect to gametocyte carriage (defined as the appearance of gametocytes on days 2–28 among those without gametocytes on day 0): 8.3% (n = 12/145) compared to 6.1% (n = 6/99), respectively, although the pattern were consistent with results of another study of lower gametocytes carriage in LPV/r group [33].

### Pharmacokinetic properties

#### Lumefantrine and desbutyl-lumefantrine

**Fig. 2:**
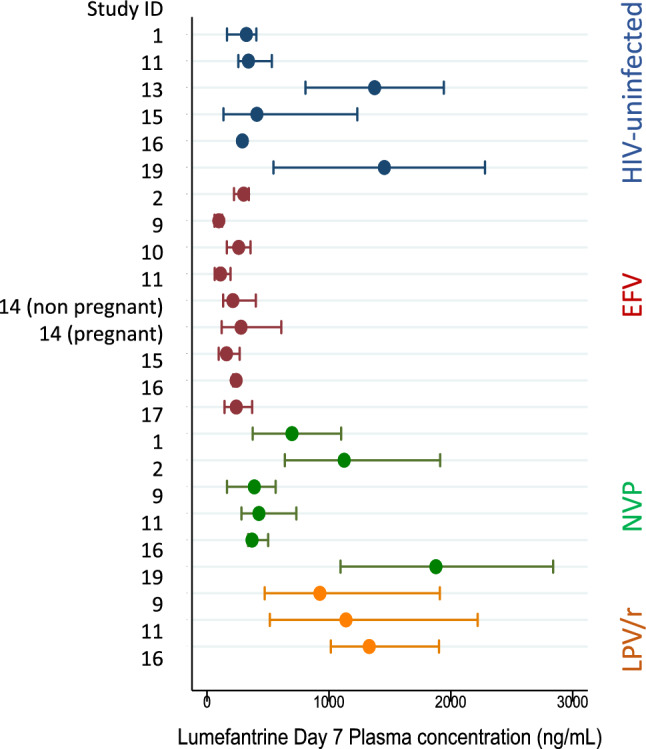
Day 7 lumefantrine concentration in PLHIV treated with different antiretroviral therapies and in HIV-uninfected patients. *EFV* efavirenz-based antiretroviral therapy, *NVP* nevirapine-based antiretroviral therapy, *LPV/r* lopinavir/ritonavir-based antiretroviral therapy. Study reported central tendency measure (mean or median or geometric mean) and estimated interquartile range are presented (for details of calculation see methods)

Eleven studies provided lumefantrine concentrations measured on day 7 for different ART regimens (Additional File 7**, **Fig. 2). Highest day 7 concentrations were observed in patients treated with LPV/r (Fig. 2). The pooled estimate of the weighted ratio between lumefantrine concentration geometric mean in patients treated with LPV/r compared to EFV was 7.89 (95%CI 6.57–9.50, p < 0.001, I^2^ = 77.6%, chi-square test for heterogeneity p = 0.011, 3 studies); and between LPV/r and NVP it was 2.83 (95%CI 2.34–3.41, p < 0.001, I^2^ = 34.8%, chi-square test for heterogeneity p = 0.216, 3 studies).

Lowest day 7 lumefantrine concentrations were observed in patients treated with EFV. The pooled weighted geometric mean ratio of lumefantrine concentration comparing NVP and EFV was estimated as 2.37 (95%CI 2.05–2.73, p < 0.001, I^2^ = 95.1%, chi-square test for heterogeneity p < 0.001, 3 studies).

Day 7 lumefantrine concentration in patients treated with NVP was also higher compared to HIV-uninfected patients with a pooled ratio of geometric mean 1.31 (95%CI 1.16–1.47, p < 0.001, I^2^ = 27.6%, chi-square test for heterogeneity p = 0.246, 4 studies), while patients treated with EFV had lower concentrations compared to HIV-uninfected patients with a pooled ratio of geometric mean 0.76 (95%CI 0.81–0.70, p < 0.001, I^2^ = 96.2%, chi-square test for heterogeneity p < 0.001, 3 studies).

Studies (Table 6) which reported lumefantrine elimination half-life in PLHIV showed that lumefantrine clearance was faster when treated with EFV compared to other ART regimens (ID 11), to TS prophylaxis only (ID 10) or not yet on treatment (ID 15). Lumefantrine half-life was shorter by 30–60% (median [IQR] 23.7 h [21.8–46.0] vs. 64.3 h [52.0–120.6], p < 0.0001 in study ID 11 [26]: the geometric mean was [90%CI] 59.2 h [46.7, 75.1] vs. 89.5 h [75.3, 106.3], p = 0.033 in study ID 15 [28], and the mean was [95% CI] 33 h [30.9–35.7] vs. 36 h [34.1–37.9], p = 0.036 in study ID 10 [37].

**Table 6:** Summary of the reported lumefantrine AUC and elimination half-life

Study ID	Statistics	HIV-uninfected group	TS prophylaxis	NVP	EFV	LPV/r	ATV/r	PLHIV not yet on ART
Lumefantrine half-life (h)								
10	Mean (95%CI)		36.0 (34.1–37.9)	33.3 (30.9–35.7)	33.3 (30.9–35.7)			
11	Median (IQR)			63.4 (46.8–111.1)	23.7 (21.8–46.0)	98.7 (88.4–119.1)		64.3 (52.0–120.6)
13	Mean (SEM)	31.16 (1.86)					42.59 (3.77)	
15	GM (90%CI)				59.2 (46.7–75.1)			89.5 (75.3–106.3)
Lumefantrine AUC (ng.h/ml)								
2^1^	Median (IQR), AUC_0-∞_			977,645 (688,477–1,383,975)	303,130 (211,080–431,962)			784,830 (547,405–1,116,250)
10	Mean (95%CI) AUC_0-∞_		375,200 (349,700–400,700)	264,800 (243,100–286,500)	264,800 (243,100–286,500)			
11	GM (90%CI), AUC_0-∞_			278,000 (228,000–339,000)	130,000 (107,000–157,000)	579,000 (477,000–704,000)		270,000 (232,000–313,000)
13	Mean (SEM), AUC_0–168_	447,976 (± 80,887)					670,530 (± 157,173)	
15	GM (90%CI), AUC_0-∞_				188 (125–281)			287 (237–349)
16	Mean (SEM), AUC_0–168_	83,508 (± 5361)		125,285 (± 35,221)	58,396 (± 8019)	357,295 (± 5156)		

*ART* antiretroviral therapies, *ATV/r* atazanavir-ritonavir, *AUC *area under the curve (from 0 h to infinite or to 168 h), *CI* confidence interval (90% or 95%), *EFV* efavirenz, *IQR* interquartile range, *GM* geometric mean, *LPV/r* lopinavir/ritonavir, *NVP* nevirapine, *PLHIV* people living with HIV, *SEM* standard error of the mean, *TS* trimethoprim-sulfamethoxazole preventive treatment

^a^Population PK study, values presented are from 9960 simulations

Area under the curve (AUC) comparison between patients on different ART regimens or TS prophylaxis was reported in 6 studies (Table 6). Compared to HIV-uninfected patients (ID 16) or to PLHIV not yet on treatment (ID 11), increased AUC was observed in PLHIV treated with LPV/r (ratio 4.3 and 2.1, respectively). AUC was decreased in PLHIV treated with EFV compared to those not yet on treatment (ratio 0.5 in study ID 11 and 0.7 in study ID 15), and similar in patients treated with NVP (ratio 1, ID 11). Also, simulations after the population pharmacokinetic modelling in study ID 2 estimated the ratio of median lumefantrine AUC for EFV-treated and NVP-treated patients to the PLHIV but ART-naïve to be 0.4 and 1.2, respectively [38].

#### Piperaquine

Only one study (ID 4) assessed the pharmacokinetics of piperaquine in PLHIV with uncomplicated *P. falciparum* malaria. In the population pharmacokinetic model of 218 malaria episodes in children from Uganda, no evidence of a significant effect on any clearance or volume distribution parameters was observed for prophylactic treatment with TS (n = 41), PLHIV not yet on ART (n = 12) nor antiretroviral therapy (n = 10) [39]. No quantitative results were provided.

#### Artemether and dihydroartemisinin (DHA)

Three studies (ID 1, ID 11, ID 15) that investigated pharmacokinetic parameters in PLHIV also included HIV-uninfected patients; these studies evaluated artemether and DHA AUC, maximum concentration (C_max_) and time to maximum concentration (T_max_). For both artemether and DHA, C_max_ and AUC_0–8_ were consistently lower in PLHIV compared to uninfected patients (Table 7), although the differences were not significant for the LPV/r group. The ratio of C_max_ and AUC_0–8_ after the first and last dose was studied in a subset of patients in the study by Kajubi et al*.* [34], and while the decrease in geometric means for artemether was observed for HIV-uninfected patients as well as for LPV/r and NVP but not EFV-treated PLHIV, the increase in values for DHA were only observed in HIV-uninfected patients and not in patients on ART.

**Table 7:** Effect of various ART regimens on DHA and artemether concentration levels, measured after the last AL dose

	Ratio (95% CI) of the geometric mean for PLHIV on various ART regimens to the geometric mean for HIV-uninfected patients		
	EFV*	NVP**	LPV/r***
Artemether AUC_0–8_	0.43 (0.30–0.62)^a^	0.34 (0.25–0.46)^e^	0.75 (0.53–1.05)
Artemether C_max_	0.46 (0.30–0.69)^b^	0.34 (0.23–0.45)^f^	0.75 (0.50–1.11)
DHA AUC_0–8_	0.37 (0.28–0.51)^c^	0.70 (0.55–0.88)^g^	0.81 (0.59–1.10)
DHA C_max_	0.40 (0.28–0.58)^d^	0.66 (0.50–0.87)^h^	0.83 (0.57–1.19)

*ART* antiretroviral therapies, *AUC* area under the curve (from 0 to 8 h), *CI* confidence interval (95%), *C*_*max*_ maximum concentration, *DHA* dihydroartemisinin, *EFV* efavirenz, *LPV/r* lopinavir/ritonavir, *CI* confidence interval, *PLHIV* people living with HIV

^*^shows results for the combined studies (ID 11, ID 15)

^**^shows results for the combined studies (ID 1, ID 11)

^***^shows results from study ID 11

^a^I-squared = 0.0%, p-value for heterogeneity = 0.150

^b^I-squared = 0.0%, p-value for heterogeneity = 0.474

^c^I-squared = 81.0%, p-value of heterogeneity = 0.022

^d^I-squared = 0.0%, p-value for heterogeneity = 0.322

^e^I-squared = 51.7%, p-value for heterogeneity = 0.150

^f^I-squared = 43.0%, p-value for heterogeneity = 0.185

^g^I-squared = 0.0%, p-value of heterogeneity = 0.445

^h^I-squared = 0.0%, p-value for heterogeneity = 0.842

No differences in time to maximum DHA concentration were detected between PLHIV treated with NVP, EFV or LPV/r compared to HIV-uninfected patients [26, 40]. For artemether, in one study (ID 1), T_max_ was observed significantly earlier (p = 0.028) in NVP (1.3 h) compared to historical controls (2 h) which was not in agreement with findings of the other study (ID 11), where a trend of rather later T_max_ (median 2.1 NVP, EVP and 3.0 in LPV/r) in PLHIV was observed compared to HIV-uninfected (2 h).

### Assessment of bias

The risk of bias in individual studies was generally considered to be low to moderate across all the domains considered for seven RCTs (Additional Files 3 and 4). In all RCTs, assessment of outcome (parasite or gametocyte positivity, drug concentration) was blinded as it was evaluated in the laboratory, independently from the clinical team. Of the 12 non-randomized studies, 11 were considered to be at low-moderate risk of bias across participant selection, intervention classification, selective reporting and outcome measurement domains. Only 3 studies were considered to be at low risk of bias due to confounding, while the remaining 9 studies were at moderate-high risk.

### Certainty of evidence

There were a couple of limitations in the few studies conducted. Firstly, a comparison of the risk of recurrence between PLHIV on TS prophylaxis and HIV-uninfected patients was performed based on total number of patients in each treatment arm and ignoring the losses to follow-up. Secondly, comparison of lumefantrine day 7 concentrations was based on the mean and standard error of the log-transformed concentration values, which for many studies were estimated from median and interquartile range. However, for three analyses for which evidence synthesis was possible, indirectness, imprecision and inconsistency were classified as not serious. The publication bias is low possibly because of the limited number of studies being conducted in patients co-infected with malaria and HIV. When all these assessments are taken together, the certainty of evidence generated from this review is likely to be low-moderate based on the GRADE guidelines [18].

## Discussion

The main finding of this systematic review is the paucity and heterogeneity of studies set in Africa comparing the efficacy of ACT in PLHIV, either untreated, on TS prophylaxis or under different ART regimens, with HIV-uninfected persons.

The only study (ID 12) looking at malaria efficacy after receiving AL or SP in PLHIV but treatment-naive adult patients [24] didn’t allow for the evaluation of efficacy of individual anti-malarial drug and failed to show any differences between PLHIV and HIV-uninfected groups in terms of parasite and gametocyte clearance, malaria recrudescence, recurrence, or reinfection on day 45. A UNAIDS report from 2022 estimated that 78% of PLHIV receive HIV treatment in sub-Saharan Africa with substantial differences in access to treatment within countries, including in children living with HIV [41]. It is however unlikely to see new studies looking at anti-malarial drug efficacy in PLHIV ART-naïve, as the latest WHO guidelines encourage rapid treatment of newly diagnosed HIV patients [42]. Therefore, delaying ART initiation until completion of malaria treatment, usually day 28 or day 42 [14] may be unethical.

Seven of the eleven studies reporting late treatment failure included HIV-uninfected participants. Risk of malaria recurrence was the most often reported outcome and the results were different for children and adults. In Uganda, where TS prophylaxis has been shown to reduce the risk of new malaria infections [43, 44], risk of recurrence was lower in children living with HIV on TS prophylaxis (with or without ART regimen) when receiving AL or ASAQ for their malaria episode [29, 30, 45]. Further studies conducted in Uganda have found that HIV-uninfected children receiving AL had significantly increased odds of malaria recurrence on day 28 compared to children living with HIV under any of the 3 ART regimens proposed (LPV/r, NVP, or EFV), [26]. The two studies conducted in adults (one each in Uganda and Nigeria) reported higher proportion (statistically non-significant) of recurrence in PLHIV on EFV or NVP compared to HIV-uninfected patients. These findings are in line with previous reviews reporting that differences in response to treatment against malaria in adults living with HIV in endemic areas may be explained by impaired acquired immunity [8, 46].

A longer parasite clearance was observed in Ugandan children when AL or DP was administered in the presence of EFV, NVP, LPV/r or TS [34, 35], a finding consistent with another report showing that children living with HIV have slower parasite clearance compared to those HIV-uninfected [34, 35, 47]. Additionally, the proportion of children living with HIV treated with LPV/r, EFV or NVP who developed gametocytes was higher than that in HIV-uninfected patients. An increased gametocyte carriage coupled with a slower parasite clearance in PLHIV on ART regimen is concerning as this population may serve as an unwitting reservoir for transmission [48] and subsequent spread of resistant parasites [49]. Better understanding the role of a weakened immune status due to HIV infection in the emergence of anti-malarial drug-resistant parasites is one of the recommendations suggested by the WHO in its strategic report to tackle this threat [50].

Drug-drug interaction between LPV/r-, NVP-, or EFV-based regimen and AL has been described in PLHIV without malaria [10, 51, 52] or in healthy adults [53]. Both nevirapine and efavirenz induce the cytochrome P450 enzyme system, which metabolizes artemether and lumefantrine, while lopinavir and ritonavir inhibit the system, which may result in an increased artemether and lumefantrine plasma concentration. Pharmacokinetic data extracted from studies considered in this review confirms these results. Comparison of different ART regimens showed a threefold increased risk of recurrence among children living with HIV on NVP or EFV compared to those on LPV/r, but also showed this effect disappeared once day 7 lumefantrine concentration was accounted in the analysis. Day 7 lumefantrine concentrations were highest in patients treated with LPV/r and lowest in patients treated with EFV, including when compared to HIV-uninfected patients (two studies) or PLHIV not yet on ART (one study). In addition, for PLHIV on EFV-based regimen, clearance of lumefantrine was significantly faster compared to that in HIV-uninfected patients; therapeutic lumefantrine concentration level of 280 ng/mL was not reached by half of the patients in one study (ID 14) and the median or mean plasma concentration level in 3 out of the 4 other studies was below that threshold. PLHIV on NVP had a similar AUC and a higher lumefantrine plasma concentration on day 7 compared to that of HIV-uninfected patients and a median concentration above the therapeutic level. PLHIV treated with a standard 3-day anti-malarial treatment for uncomplicated *P. falciparum* malaria may contribute between 2.6 and 6.6% of estimated excess failures because of suboptimal anti-malarial drug dosing [3]. Lower exposure to lumefantrine, especially in PLHIV on EFV is of concern as it could not only result in treatment failure but also contribute to the spread of resistant parasites. The poor efficacy of AL in PLHIV on EFV has already been described [54, 55]. Prolonging the duration of the AL treatment from 3 to 5 days could be considered as it would reduce the risk of having a day 7 lumefantrine plasma concentration below the therapeutic threshold [55]. Malaria patients co-infected with HIV are considered a special risk group in the WHO malaria treatment guidelines [12]; however, to date, no dose adjustment for this risk group has been officially endorsed. The WHO now recommends the use of dolutegravir-based ART regimen as first line treatment for PLHIV and EFV-based regimen as an alternative first-line regimen; dolutegravir has fewer drug-drug interactions and does not seem to modify AL pharmacokinetics [56, 57].

This review is limited by the minimal meta-analyses performed due to differences in the presentation of the data and the reported estimates.

## Conclusion

Limited data on ACT treatment outcomes or drug exposure in PLHIV in Africa remains a reality to date, and there is important heterogeneity in study designs limiting the interpretation of the results. PLHIV on EFV appears nevertheless to be at risk of suboptimal dosing when treated with a standard 3-day AL regimen for uncomplicated *P. falciparum* malaria and also at a higher risk of treatment failure. Conducting an individual patient data meta-analysis to explore the impact of antiretroviral therapy on anti-malarial treatment would help understand these complex interactions better.

## Supplementary Information

Additional file 1. Search terms for the original search in MEDLINE, EMBASE, Web of Science, Cochrane Central, WHO Global Index Medicus and Clinicaltrials.gov

Additional file 2. Search terms for the subsequent searches in the WWARN Clinical Trial Library

Additional file 3. ROB assessment for non-RCTs: methods and outline of the risk of bias tables and responses to each of the signalling questions

Additional file 4. ROB assessment for RCTs: methods and outline of the risk of bias tables and responses to each of the signalling questions

Additional file 5. Inclusion criteria for enrolment of people living with HIVin each study included in the review and their baseline CD4 measurements

Additional file 6. Geographical distribution of the 19 study sites and their malaria transmission level as reported in individual manuscripts

Additional file 7. Summary of the reported day 7 lumefantrine concentrations

## Data Availability

All data generated or analysed during this study are included in this published article and its supplementary information files.

## Abbreviations

ACT: Artemisinin-based combination therapy
AL: Artemether-lumefantrine
AP: Artesunate-pyronaridine
ART: Antiretroviral therapy
ASAQ: Artesunate-amodiaquine
ASMQ: Artesunate-mefloquine
ASSP: Artesunate-sulfadoxine-pyrimethamine
ATVr: Atazanavir-ritonavir
AUC: Area under the curve
CI: Confidence interval
C_max_: Maximum concentration
DHA: Dihydroartemisinin
DP: Dihydroartemisinin-piperaquine
EFV: Efavirenz
HAART: Highly active antiretroviral therapy
HIV: Human immunodeficiency virus
HR: Hazard ratio
IQR: Interquartile range
KM: Kaplan–Meier
LPV/r: Lopinavir-ritonavir
LUM: Lumefantrine
N/A: Non available
NVP: Nevirapine
OR: Odds ratio
PCR: Polymerase chain reaction
PK: Pharmacokinetics
PLHIV: People living with HIV
PPQ: Piperaquine
RCT: Randomized clinical trial
RR: Risk ratio
SP: Sulfadoxine-pyrimethamine
T_max_: Time to maximum concentration
TS: Trimethoprim-sulfamethoxazole
WHO: World Health Organization
WWARN: WorldWide antimalarial resistance network
