# Supplementary figures and images for: Efficacy of artemisinin-based combination therapy (ACT) in people living with HIV (PLHIV) diagnosed with uncomplicated Plasmodium falciparum malaria in Africa: a WWARN systematic review
Source: Malar J. 2025 May 16;24:153. doi: 10.1186/s12936-025-05393-8 (PMC12083008; doi:10.1186/s12936-025-05393-8)

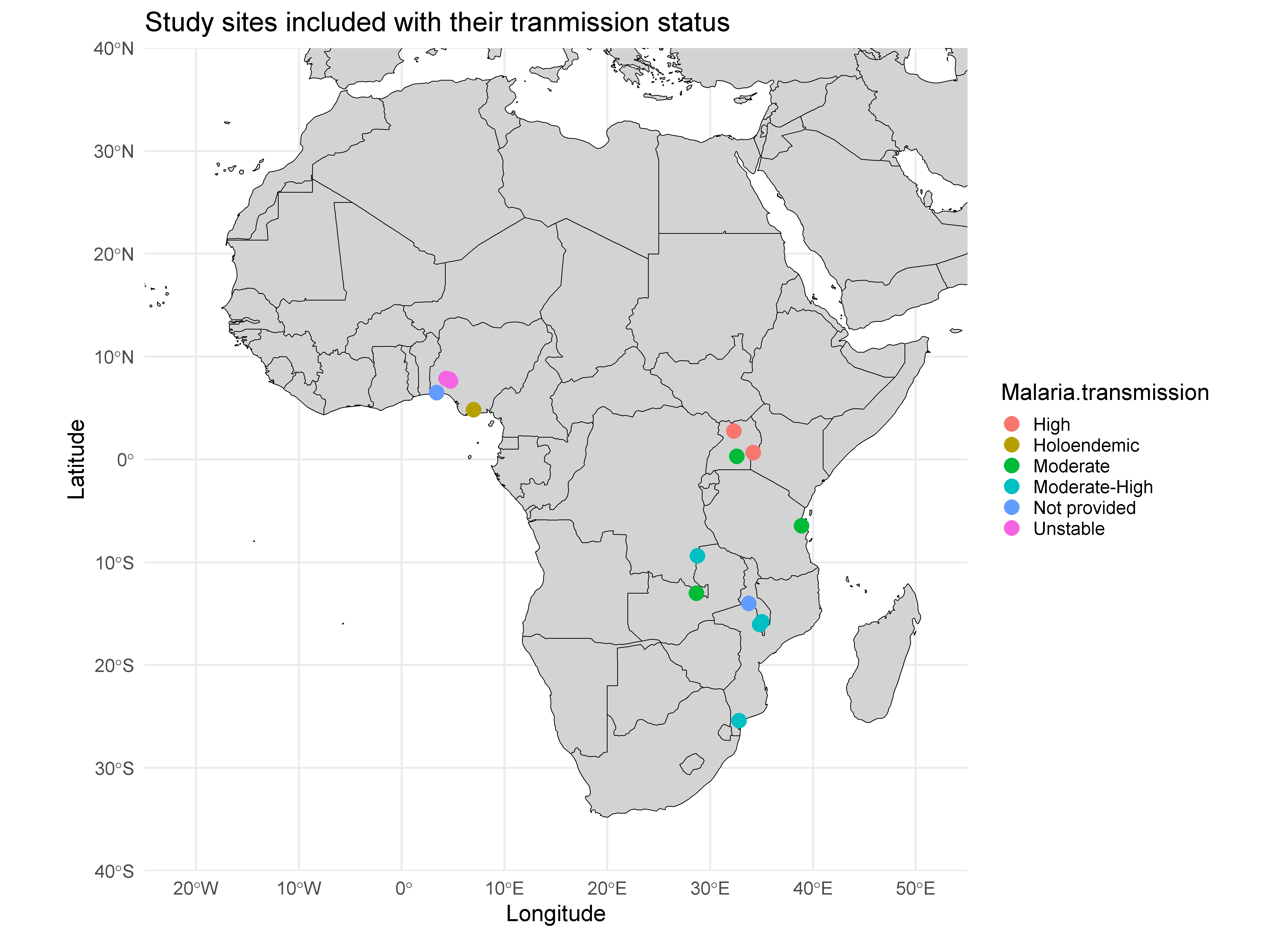

Supplement: Supplementary file 6 — Additional file 6. Geographical distribution of the 19 study sites and their malaria transmission level as reported in individual manuscripts [file 12936_2025_5393_MOESM6_ESM.tiff]
