# Supplementary material for: Efficacy of artemisinin-based combination therapy (ACT) in people living with HIV (PLHIV) diagnosed with uncomplicated Plasmodium falciparum malaria in Africa: a WWARN systematic review
Source: Malar J. 2025 May 16;24:153. doi: 10.1186/s12936-025-05393-8 (PMC12083008; doi:10.1186/s12936-025-05393-8)
Supplement: Supplementary file 7 — Additional file 7. Summary of the reported day 7 lumefantrine concentrations [file 12936_2025_5393_MOESM7_ESM.docx]

**Additional File 7. Summary of the reported day 7 lumefantrine concentrations (ng/mL)**

| Study ID | Publication reference | HIV treatment | N | Specific Group | Median | Geometric Mean | Mean | SEM | IQR | Range | 95%CI | 90%CI | N below 280 ng/mL (%) |
| --- | --- | --- | --- | --- | --- | --- | --- | --- | --- | --- | --- | --- | --- |
| 2 | [1] | EFV | N/A |  | 300.4 |  |  |  | 220.8- 343.1 |  |  |  |  |
| 9 | [2] | EFV | 25 |  | 97 |  |  |  | 61-124 |  |  |  |  |
| 10 | [3] | EFV+TS | 22 |  |  |  | 260 |  | 163-357 ^5^ |  | 200-320 |  |  |
| 11 | [4] | EFV | 48 |  | 111 |  |  |  | 63-192 |  |  |  |  |
| 9 | [2] | LPV/r | 65 |  | 926 |  |  |  | 473-1910 |  |  |  |  |
| 11 | [4] | LPV/r | 70 |  | 1140 |  |  |  | 515-2220 |  |  |  |  |
|  |  | HIV-uninfected group | 186 |  | 340 |  |  |  | 257-531 |  |  |  |  |
| 1 | [5] | HIV-uninfected group | 20 |  | 323 |  |  |  | 164- 404 ^4^ | 53.9-779 |  |  |  |
| 2 | [1] | None | N/A |  | 970 |  |  |  | 562.1-1729 |  |  |  |  |
| 1 | [5] | NVP | 15 |  | 697 |  |  |  | 374-1100 ^4^ | 155-2250 |  |  |  |
| 2 | [1] | NVP | N/A |  | 1125 |  |  |  | 638.8-1913 |  |  |  |  |
| 9 | [2] | NVP | 67 |  | 388 |  |  |  | 164-563 |  |  |  |  |
| 11 | [4] | NVP | 62 |  | 426 |  |  |  | 282-733 |  |  |  |  |
| 10 | [3] | TS | 22 |  |  |  | 640 |  | 479-801 ^5^ |  | 540-740 |  |  |
| 13 | [6] | ATV/r | 10 |  |  |  | 3847 | 894 |  |  | 2096-5598 ^5^ |  |  |
|  |  | HIV-uninfected group | 10 |  |  |  | 1375 | 266 |  |  | 854-1896 ^5^ |  |  |
| 14 | [7] | EFV | 27 | Pregnant women | 279 |  |  |  | 120-610 |  |  |  |  |
|  |  | EFV | 25 | Non-pregnant women | 212 |  |  |  | 133-400 |  |  |  |  |
| 14 | [8] | EFV | 11 | NR1I3 1089 TT* | 146.1 |  |  |  |  |  | 83.6-313.2 |  | 70 |
|  |  | EFV | 42 | NR1I3 1089 TC and CC* | 271.1 |  |  |  |  |  | 144.7-479.6 |  | 53 |
|  |  | EFV | 20 | CYP2B6 516 GG* | 289.4 |  |  |  |  |  | 148.1-569.9 |  | 47 |
|  |  | EFV | 33 | CYP2B6 516 TT and GT* | 211.7 |  |  |  |  |  | 123.1-352 |  | 59 |
| 15 | [9] | HIV-uninfected group | 30 | Pregnant women |  | 409 |  |  |  |  |  | 231-617 |  |
|  |  | EFV | 9 | Pregnant women |  | 160 |  |  |  |  |  | 134-309 |  |
| 16 | [10] | None | 10 |  | 290 |  |  |  | 275-304 |  |  |  |  |
|  |  | NVP | 10 |  | 369 |  |  |  | 337-502 |  |  |  |  |
|  |  | EFV | 10 |  | 239 |  |  |  | 213-243 |  |  |  |  |
|  |  | LPV/r | 10 |  | 1331 |  |  |  | 1015-1903 |  |  |  |  |
| 17 | [11] | EFV | 85 ^1^ |  | 240 |  |  |  | 143.2-370 |  |  |  |  |
| 19 | [12] | HIV-uninfected group | 94 |  | 1455 ^2^ |  |  |  | 545-2280 |  |  |  |  |
|  |  | NVP | 68 |  | 1878 ^3^ |  |  |  | 1095-2840 |  |  |  |  |

*patients grouped according to cytochromes P450: CYP2B6 516 GG, CYP2B6 516 GT and CYP2B6 516 TT genotypes and nuclear receptors NR1I3 152c-1089: TC and TT alleles

^1^ an additional 36 patients were below the lower limit of quantification (<50 ng/mL)

^2^ reported as 2.75 (IQR 1.03-4.31) µM, a molecular weight of 528.939 g/mol was used in the calculation; there were also 5 participants with extremely low readings not included in the calculation

^3^ reported as 3.55 (IQR 2.07-5.37) µM, a molecular weight of 528.939 g/mol was used in the calculation

^4^ estimated using the methods described by Hozo, *et al*. [13]

^5^ calculated using <http://vassarstats.net/median_range.htm>

ATV/r: atazanavir-ritonavir; CI: confidence interval (90 or 95%); EFV: efavirenz; IQR: interquartile range; LPV/r: lopinavir-ritonavir; NVP: nevirapine; N/A: information not available; SEM: standard error of the mean; TS: trimethoprim-sulfamethoxazole preventive treatment

Study ID: studies included in the review and labelled as in Table 1 of the main manuscript

**References**

1. Maganda BA, Minzi OM, Kamuhabwa AA, Ngasala B, Sasi PG. Outcome of artemether-lumefantrine treatment for uncomplicated malaria in HIV-infected adult patients on anti-retroviral therapy. Malar J. 2014;13:205.

2. Achan J, Kakuru A, Ikilezi G, Ruel T, Clark TD, Nsanzabana C, et al. Antiretroviral agents and prevention of malaria in HIV-infected Ugandan children. N Engl J Med. 2012;367:2110-8.

3. Musoke D, Bergmann TK, Ntale M, Sodemann M, Ogwal-Okeng J. Pharmacokinetics of lumefantrine in adults co-infected with malaria and HIV-1: with and without efavirenz-based antiretroviral therapy. Int J Trop Med. 2012;7:187-92.

4. Parikh S, Kajubi R, Huang L, Ssebuliba J, Kiconco S, Gao Q, et al. Antiretroviral choice for HIV impacts antimalarial exposure and treatment outcomes in Ugandan children. Clin Infect Dis. 2016;63:414-22.

5. Huang L, Carey V, Lindsey JC, Marzan F, Gingrich D, Graham B, et al. Concomitant nevirapine impacts pharmacokinetic exposure to the antimalarial artemether-lumefantrine in African children. PLoS One. 2017;12:e0186589.

6. Usman SO, Oreagba IA, Kadri MR, Adewumi OO, Akinyede A, Agbaje EO, et al. Evaluation of the effects of atazanavir-ritonavir on the pharmacokinetics of lumefantrine in patients living with HIV in Lagos University Teaching Hospital, South-Western Nigeria. Eur J Clin Pharmacol. 2021;77:1341-8.

7. Adegbola A, Abutaima R, Olagunju A, Ijarotimi O, Siccardi M, Owen A, et al. Effect of pregnancy on the pharmacokinetic interaction between efavirenz and lumefantrine in HIV-malaria coinfection. Antimicrob Agents Chemother. 2018;62:e01252-18.

8. Adegbola AJ, Rana A, Adeagbo BA, Bolarinwa RA, Olagunju AE, Siccardi M, et al. Influence of selected polymorphisms in disposition genes on lumefantrine pharmacokinetics when coadministered with efavirenz. Pharmacogenet Genomics. 2020;30:96-106.

9. Hughes E, Mwebaza N, Huang L, Kajubi R, Nguyen V, Nyunt MM, et al. Efavirenz-based antiretroviral therapy reduces artemether-lumefantrine exposure for malaria treatment in HIV-infected pregnant women. J Acquir Immune Defic Syndr. 2020;83:140-7.

10. Usman SO, Oreagba IA, Akinyede AA, Agbaje EO, Akinleye MO, Onwujuobi AG, et al. Effect of nevirapine, efavirenz and lopinavir/ritonavir on the therapeutic concentration and toxicity of lumefantrine in people living with HIV at Lagos University Teaching Hospital, Nigeria. J Pharmacol Sci. 2020;144:95-101.

11. Banda CG, Chaponda M, Mukaka M, Mulenga M, Hachizovu S, Kabuya JB, et al. Efficacy and safety of artemether-lumefantrine as treatment for *Plasmodium falciparum* uncomplicated malaria in adult patients on efavirenz-based antiretroviral therapy in Zambia: an open label non-randomized interventional trial. Malar J. 2019;18:180.

12. Chijioke-Nwauche I, van Wyk A, Nwauche C, Beshir KB, Kaur H, Sutherland CJ. HIV-positive nigerian adults harbor significantly higher serum lumefantrine levels than HIV-negative individuals seven days after treatment for *Plasmodium falciparum* infection. Antimicrob Agents Chemother. 2013;57:4146-50.

13. Hozo SP, Djulbegovic B, Hozo I. Estimating the mean and variance from the median, range, and the size of a sample. BMC Med Res Methodol. 2005;5:13.
